# Data challenges of biomedical researchers in the age of omics

**DOI:** 10.7717/peerj.5553

**Published:** 2018-09-11

**Authors:** Rolando Garcia-Milian, Denise Hersey, Milica Vukmirovic, Fanny Duprilot

**Affiliations:** 1 Bioinformatics Support Program, Research and Education Services, Cushing/Whitney Medical Library, Yale University, New Haven, CT, United States of America; 2 Science Libraries, Lewis Science Library, Princeton University, Princeton, NJ, United States of America; 3 Pulmonary Critical Care & Sleep Medicine, Yale School of Medicine, Yale University, New Haven, CT, United States of America; 4 Service commun de la documentation, Université Denis Diderot (Paris VII), Paris, France

**Keywords:** Information seeking behavior, Data interpretation, Statistical, Genomics, Computational biology, Software, Survey

## Abstract

**Background:**

High-throughput technologies are rapidly generating large amounts of diverse omics data. Although this offers a great opportunity, it also poses great challenges as data analysis becomes more complex. The purpose of this study was to identify the main challenges researchers face in analyzing data, and how academic libraries can support them in this endeavor.

**Methods:**

A multimodal needs assessment analysis combined an online survey sent to 860 Yale-affiliated researchers (176 responded) and 15 in-depth one-on-one semi-structured interviews. Interviews were recorded, transcribed, and analyzed using NVivo 10 software according to the thematic analysis approach.

**Results:**

The survey response rate was 20%. Most respondents (78%) identified lack of adequate data analysis training (e.g., R, Python) as a main challenge, in addition to not having the proper database or software (54%) to expedite analysis. Two main themes emerged from the interviews: personnel and training needs. Researchers feel they could improve data analyses practices by having better access to the appropriate bioinformatics expertise, and/or training in data analyses tools. They also reported lack of time to acquire expertise in using bioinformatics tools and poor understanding of the resources available to facilitate analysis.

**Conclusions:**

The main challenges identified by our study are: lack of adequate training for data analysis (including need to learn scripting language), need for more personnel at the University to provide data analysis and training, and inadequate communication between bioinformaticians and researchers. The authors identified the positive impact of medical and/or science libraries by establishing bioinformatics support to researchers.

## Background

With the development of lower cost of high-throughput technologies (e.g., next generation sequencing, microarrays, mass spectrometry), biomedical researchers can now generate unprecedented amounts of diverse omics data. We use omics as defined by Horgan & Kenny (2011), a holistic view or universal detection of the molecules (e.g., genes, genomics; mRNA, transcriptomics; proteins, proteomics) in a specific biological sample (Horgan & Kenny, 2011). While this advancement allows researchers faster access to data, it created a gap between data production and analysis that tremendously slows publishing study results. Data analysis became a major bottleneck for researchers, since it requires very specialized training and the use of dedicated bioinformatics software tools (Carvalho & Rustici, 2013; Geskin et al., 2015). Very often data analysis requires collaboration amongst researchers, clinicians, statisticians and bioinformaticians (Klupczynska, Derezinski & Kokot, 2015). Thus, a delay in omics and high-throughput data (high-throughput data defined as those obtained from the use of high-throughput methodologies such as next generation sequencing, microarray, mass spectrometry, etc.) interpretation results on more time needed to finalize projects based on -omics methodology (Alyass, Turcotte & Meyre, 2015; Gligorijevic, Malod-Dognin & Przulj, 2016; Merelli et al., 2014).

For bench biomedical researchers who wish to perform data analysis independently, with minimal assistance of a statistician (and/or bioinformatician) or without one, one of the greatest challenges is understanding how to use necessary computational tools (van Kampen & Moerland, 2016). Most bench researchers have not been trained to use the bioinformatics resources available to analyze high-throughput data, and perhaps more significantly, they do not have enough time to be trained in statistics or bioinformatics (Carvalho & Rustici, 2013). Many medical and health science libraries are helping researchers to overcome these challenges.

Medical Libraries in the United States have been providing bioinformatics services to support biomedical research since 1990 (Owen, 1996; Pratt, 1990); however, formal library-centered bioinformatics programs began to appear only after the year 2000. These programs usually include consultation services, education, training, and networked biological information resources (Yarfitz & Ketchell, 2000) with access to commercial bioinformatics software (Chattopadhyay et al., 2006). More recently, in-depth data analysis services for researchers were included in a program and resulted in collaborative studies between librarians and faculty members (Li, Chen & Clintworth, 2013) published in peer reviewed journals. 

Similarly, in 2014, the Cushing Whitney Medical Library at Yale University started developing a Bioinformatics Support Program intended to support Yale University-affiliated biomedical researchers (Garcia-Milian, 2015, http://works.bepress.com/rolando_garciamilian/6/). In this study we aim to identify the most common challenges that researchers at Yale University still face in analyzing omics data. The results of this study provide recommendations on how to improve bioinformatics support to researchers at universities and similar academic institutions.

## Methods

This study was designed as a multimodal needs assessment analysis, combining an online survey and in-depth one-on-one semi-structured interviews. The authors chose to use both a survey and semi-structured interviews as complementary means of data collection because this approach is well suited for exploring perceptions and opinions of respondents on complex issues (Barriball & While, 1994). In addition, this method provided the authors with the opportunity to probe interviewees for more information or clarification on topics that surfaced in the survey.  

In order to ensure the safety and anonymity of the participants in this study, both the survey and the subsequent interviews were approved by the Yale University Review Board (HSC# 1511016778). On January 14, 2016, the authors emailed an invitation with a link to participate in an online survey to 860 Yale-affiliated individuals who had registered for at least one training session offered through the Cushing/Whitney Medical Library’s Bioinformatics Program.  It remained open until March 15, 2016, and contained the following sections: (1) Demographics, (2) Information/Data-seeking assessment, (3) Databases, software and tools, (4) Training and (5) Collaborative work and networking events. A copy of the survey instrument is available online: https://works.bepress.com/rolando_garciamilian/25/.

In addition, fifteen in-person interviews were arranged with volunteers who responded to the survey and were selected from the main three respondent groups of the survey: five faculties, five postdocs, and five graduate students. Interviews were conducted at a time and location chosen by the participant and were digitally audio-recorded (authors RGM/DH/FD). Each interview lasted about 30 minutes.  All participants were asked the same questions while retaining the flexibility to explore participants’ responses in further detail where necessary. The interviews are available online: https://works.bepress.com/rolando_garciamilian/26/. Interviews were transcribed by interviewers (RGM/DH) or by the Medical Library support staff, then checked for accuracy by the respective interviewer (RGM/DH/FD). Data were analyzed using NVivo 10 (RGM/DH/FD) according to the thematic analysis approach (Braun & Clarke, 2006).

## Results

### Survey

**Figure 1:**
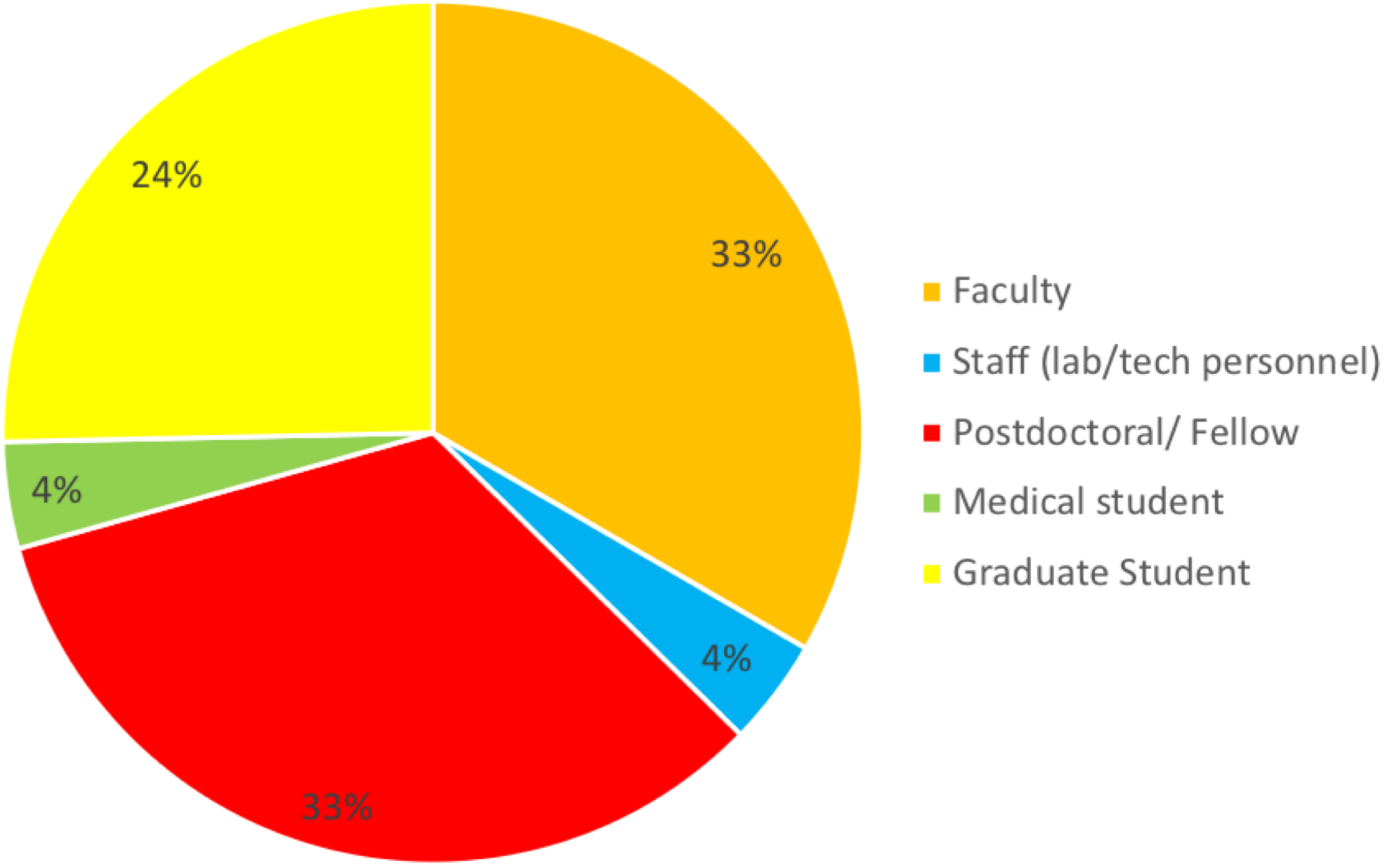
Response to the question: which of the following best describes your role? Total responses: 157.

**Figure 2:**
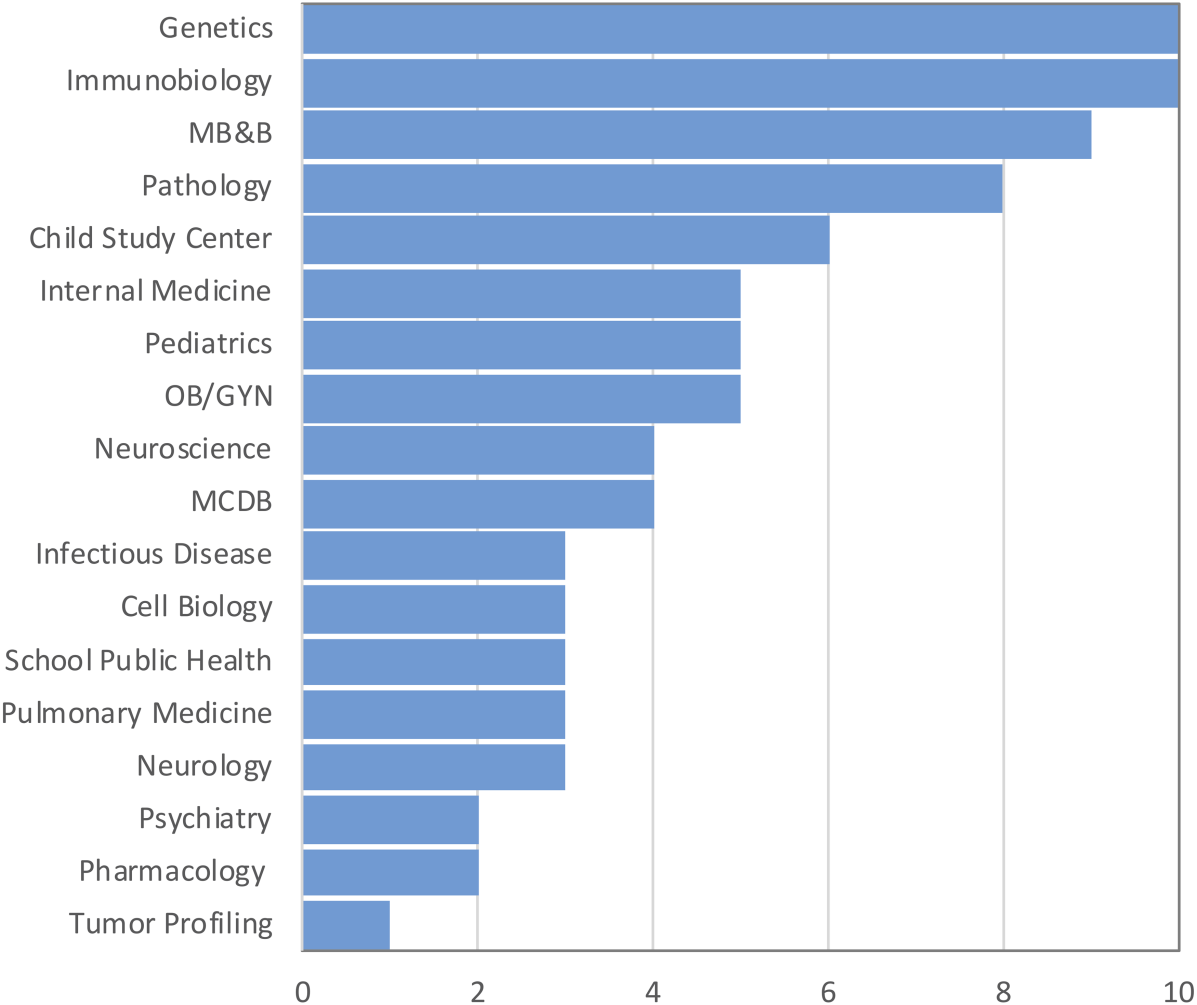
Top departments by the number of respondents. Total responses: 146.

The purpose of the survey was to identify the most common challenges researchers face in analyzing omics data. A total of 176 individuals out of 860 responded resulting in 20% response rate. Among those who indicated their position (*n* = 157), three well-defined groups of respondents received the survey: faculty (33%), postdocs (33%), and graduate students (24%) (50, 50 and 38, respectively). Six medical students and six lab technicians also responded to the survey (Fig. 1). None of the respondents identified themselves as residents or clinical fellows. Genetics and Immunobiology departments were the most highly represented (10 respondents both), followed by the Department of Molecular Biophysics and Biochemistry (MB&B) and Pathology (nine and eight respondents, respectively). The rest of departments represented can be seen in Fig. 2.

Respondents selected high-throughput data analysis (e.g., RNA-seq, microarray) as their most important type of data analysis followed by network and pathway analysis, and functional analysis of high-throughput data. Other data analysis needs included analysis of flow cytometry data, statistical analysis (e.g., meta-analysis), image analysis, and mass spectrometry data analysis among others (Table 1).

**Table 1:** Response to the question: please indicate how important are the following types of data analysis for your research. Each cell contains the number of respondents and the percentage of the total. The darker the color the higher the number of responses. Total responses: 134.

**Data analysis**	**Not important**	**Important**	**Very important**	**Total responses**
Analysis of high-throughput data (e.g., microarray data, RNA-seq)	16 (11.9%)	21 (15.7%)	97 (72.4%)	134 (100%)
Signaling, network, and pathway analysis	13 (10%)	33 (25.4%)	84 (64.6%)	130 (100%)
Functional analysis of high-throughput data	20 (15.4%)	36 (27.7%)	74 (56.9%)	130 (100%)
Transcription factor and gene regulatory sequence analysis	25 (19.1%)	38 (29.0%)	68 (51.9%)	131 (100%)
Integrated searches of literature and high-throughput data	15 (11.6%)	50 (38.8%)	64 (49.6%)	129 (100%)
DNA/protein sequence manipulation and analysis	17 (13.3%)	50 (39.1%)	61 (47.7%)	128 (100%)
SNP, genetic variation, Genome wide association data analysis	42 (31.8%)	42 (31.8%)	48 (36.4%)	132 (100%)
Other data analysis needs	11 (43.4%)	4 (12.5%)	17 (53.1)	32 (100%)

**Figure 3:**
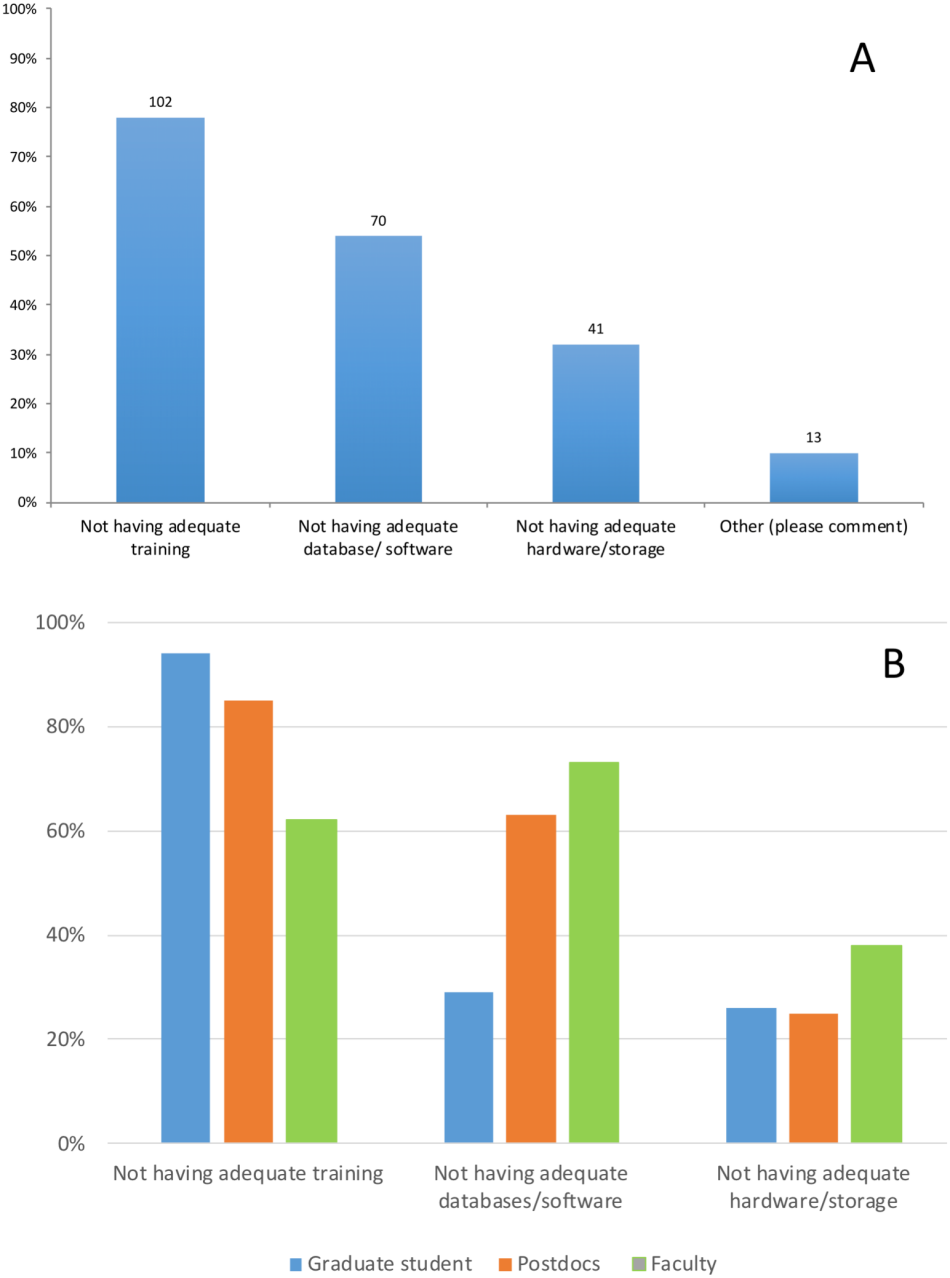
Response to the question: (A) What are the main challenges with data analysis? (B) Responses to this question by position. Total responses: 130.

When asked about main challenges with data analysis (Q.6), most respondents (78%; *n* = 130) identified lack of adequate training as the main challenge they face in their research. The next most noted challenge was not having the proper database or software (54%; *n* = 130) to expedite analysis. Under “Other,” respondents mentioned: training beyond the basics of command-line, and inadequate Information Technology (IT) support. However, not having the proper training is perceived as a bigger obstacle for graduate students and postdocs (94% and 86% of respondents) than for faculty (62%). In contrast, the main challenge for faculty is not having the adequate software, databases or tools (73%). Postdocs also identified this as a problem (62%), however not having the adequate software, databases or tools was not the main concern of graduate students (28%) (Figs. 3A 3B).

Respondents indistinctively replied affirmatively (52%; *n* = 129) or negatively (48%; *n* = 129) to the question: Does finding, retrieving, analyzing high-throughput omics-related, or other type of data represent a bottleneck for your research project? (Q.7). However, when asked: Do you have data that you have not been able to analyze? Only twenty-seven percent ( *n* = 128) responded affirmatively that this is due to the lack of proper software (Q.8) while 47% (*n* = 128) attributed it to the lack of training (Q.10). When asked about the type of training sessions needed, programming with R, Python, Perl, Unix, and Linux for the analysis of high-throughput data (e.g., microarray, RNA-sequencing) were mentioned the most. Others included Galaxy, MatLab, SPSS, and statistics for biologists (Q.11).

The majority of respondents (72%; *n* = 107) considered it helpful to have access to working groups and discussion panels on the challenges and solutions to data collection and analysis (Q.12).

### Interviews

We used Nvivo 11 Pro for Windows software (QSR International Pty Ltd) for qualitative analysis of the text to identify main challenges for data analysis reported in the interviews.

While researchers enumerated various challenges related to data analysis, two main themes emerged from the interviews; personnel and training. Researchers feel they could improve data analyses practices if they had better access to the appropriate expertise for data analyses, and/or training in data analyses tools and resources. In both cases, interviewees noted time as an underlying barrier. Researchers consistently commented on the time it takes for them or others to analyze data; difficulty in finding time to acquire expertise in using data analyses tools; and a lack of time to investigate and evaluate the resources available to help them analyze their data.

#### Personnel challenges

Among the 15 interviewees, 13 commented on challenges related to personnel. Experts in data analysis are in high demand and many labs, particularly smaller ones, have difficulty getting access to staff with those skill sets. As a result, researchers feel that valuable time is wasted either waiting to work with an expert or attempting to do the analysis with their own staff.

“[It’s] *hard to get someone to sit down and analyze the data for you. Those who are able to do it are in high demand.*” (Faculty)

“*Well you go to the person that you are with and there is a queue because the other person is using the same person. So it is based on first come, first served.*” (Faculty)

Several researchers have expressed the need to have more personnel available to analyze their data.

*“We need more people so they can do more analysis. I see, the technology generates more data but there is only one person to analyze these growing amounts of data.*” (Faculty)

“*Getting the data is not the problem these days. Interpreting that data is. It requires someone with real expertise who does this all the time.”* (Faculty)

“*It would be helpful to have someone who is a little more hands-on to help analyze the data.*” (Faculty)

Even when researchers had access to a biostatistician or bioinformatician, poor communication between these professionals was reported to threaten the quality of their collaboration. Biomedical researchers and bioinformaticians or biostatisticians do not have the same backgrounds and do not always use the same terminology in the same ways. This is particularly true if a researcher is working with a statistician with very little background in medicine or biology:

“*Then, it will be a challenge to sit down and pick up what is relevant because the bioinformatician does not always understand the biology behind it, so the challenge is really extracting what is pertinent to your project because the bioinformatician can tell you, ‘okay this is significant’ but it may be total nonsense for you.”* (Postdoc)

*“There is a little disconnect between how he did the analyses and how we understand what he did. Part of it is that they don’t do any biology, and we only do biology, so there is that difference there*” […] *I think this happens in many fields with people who work with computers and people who work with something else. You almost need a translator.*” (Graduate Student)

This divide between the researcher’s lack of statistical skills and the biostatistician’s or bioinformatician’s lack of understanding of the biological basis behind the data may result in wrongly addressing the original biomedical research question.

Some researchers commented on the desire to have access to centralized institutional support in terms of bioinformatics expertise, even when they are working to analyze the data on their own.

“*But for the Yale community there are no bioinformatic resources, people that you could talk to if you have an issue, some kind of analysist, some expert that you could refer to*.” (Postdoc).

#### Training challenges

Training is also a significant challenge to successfully analyze data. Among the 15 interviewees, 10 commented on problems related to the lack of training that would allow them to conduct their own data analysis.

In order to efficiently use the preponderance of new tools and resources, a certain level of expertise in data analysis is required. Unfortunately, researchers recognize that they do not always have a strong background in this type of work.

“*I do not have a bioinformatics background. Basically, I am a biologist. I do not go further than the interface that you see on Excel. For me having a person who trains [me] is necessary.*” (Postdoc)

“*We are not trained in our lab in bioinformatics. You can see clearly that half of the time we don’t really know what are the analysis that we are dealing with.*” (Postdoc)

“*I am graduate student and everything is new, so every time you do something, you totally have to learn how to do it. I have no background in data analysis. I was a very heavy chemistry undergrad and did not do any high throughput [work] at all. It has been a lot to try to learn the computer.*” (Graduate Student)

“*In analyzing the data, a lot of it is a lack of expertise on my part, especially when it comes to statistics, so sometimes the answers that we get are different than the questions that we originally asked.*” (Faculty)

Further investigation of researchers’ training needs identified two types. First, many interviewees commented on their desire to get training to help them identify which resources to use in data analysis and how to use them, *“because in order to use a tool, you need to know what are the steps that you are going to use, why, what to avoid, what to look for.*” (Faculty)

These researchers want to be able to do their own analysis and not rely on other staff. Many would like access to commercial bioinformatic software. Their training needs vary from learning how to use specific commercial tools such as Ingenuity Pathway Analysis (IPA), to how to do bioinformatics analysis using a high-performance computer cluster, code with R, or a desire for more general training in statistics. These needs reflect those identified from the survey as well (e.g., Q9 access to commercial bioinformatics software, Q10/11 training needs).

Several researchers have attempted to acquire training by attending classes, watching online tutorials, or enrolling in workshops. However, individuals identified various preferences for training. How best to deliver training remains a challenge since it is related to the issue of time which researchers have already identified as problematic.

“*It is mostly because we don’t have time to train in bioinformatics, not because we don’t want to.*” (Postdoc)

Moreover, the usefulness of the training depends, for some researchers, on the likelihood they will use the content of the training shortly after.

“…*you do it once with the preceptor at the podium and you know that you are going to forget if you don’t use it right away.*” (Faculty).

The interviews also identified an additional pain point with training needs. For some researchers, training is not only about how to use a tool but also about understanding how the person traditionally in charge of data analysis (a bioinformatician or a statistician) would do the work using that tool. In this case, the researcher does not plan to conduct the data analysis themselves, but are eager to interact efficiently with “core people” doing the analysis.

“*You need to know how to talk to a bioinformatician. You need to have someone know what code looks like, what optimization means [and] help researchers understand what the limitations are and what to ask bioinformaticians or statisticians. Training that would allow researchers to understand what statisticians can do, to allow them to communicate better with them, would be helpful.”* (Faculty)

This type of training would help address already identified communication problems that exist between researchers and biostatisticians as they attempt to work together.

## Discussion

### The need for training

Both survey and interview respondents identified an unmet need for training to analyze high-throughput omics data. While graduate students and postdocs acknowledged that this was their biggest obstacle, the survey results showed that this was not the main concern for faculty. Within interviews, faculty clearly stated awareness that graduate students and postdocs in their lab, who were required to do much of the data analysis, needed training opportunities, and expressed a desire for these opportunities to be offered. The need for training was not solely observed at Yale University. Recently published studies from academic medical centers also showed that respondents expressed interest in training on bioinformatic analytical tools, as well as for training support of an analytic workforce (Geskin et al., 2015; Oliver, 2017). Those who responded to our survey specified training needs such as programming or scripting language (e.g., R, Python, Perl, Linux) for the analysis of high-throughput data as the top desired type of training (Survey Question 11). As biomedical research has become more data-intensive, some believe that the ability to design and write computer programs is among the most crucial skills that a contemporary biologist should acquire and develop (Ekmekci, McAnany & Mura, 2016). However, it is important to notice that the researcher’s perceived need to learn programming and command-line may ignore the consequent investment of time needed to learn these skills.

Our study also identified one paradox, where researchers asked for training, but also reported the lack of time for attending training sessions. Bioinformatics training is challenging not only because of its interdisciplinary nature, but also because of the fast-paced changes of the required technology. Incorporation of trainees’ feedback during the designing stages of the training could result in more efficient sessions for researchers (Pavelin et al., 2012).

Researchers also recognized that bioinformatics support, available in a timely efficient manner when they encounter problems, is highly advantageous. Development of a centralized infrastructure or support at the University would be helpful, since centralization of resources was previously identified as an important institutional gap (Geskin et al., 2015). Having bioinformatics support (in addition to the services provided by the core research facilities (https://medicine.yale.edu/cores/)), similar to tech support provided by commercial bioinformatics software vendors, would be a valuable service for researchers at the University.

### Personnel challenge

The interviews revealed an issue that the survey was not able to delve into: adequate communication (or lack of) with statisticians and bioinformaticians. There is a definite need for the latter to understand some level of biological work or research, and vice versa. This misunderstanding results in time wasted and growing frustrations reflected in the interview responses. It would be beneficial for statisticians and informaticians, who work with biomedical researchers, to be trained in biological standards or terminology to improve the interaction between these staff and ensure that their time is used in a more efficient fashion. One contradiction was identified in our study where respondents stated they wanted more access to bioinformatics experts, but also complained of communication problems with them. One study describes this disconnect as the result of the absence of statisticians from the planning stage of the original research at which point any concerns or questions about the analyses could be resolved proactively (Carvalho & Rustici, 2013).

It is important to mention that previous studies have identified researchers’ concerns regarding hardware, data storage and space to analyze data (Norton et al., 2016). Our survey results identified this as a problem only for the 30% of respondents. Interviewees have not indicated concerns with data storage nor with computing storage. At Yale University, the primary and secondary stages of the data lifecycle, where big/raw data files are generated, usually takes place in core units (e.g., Yale Center for Genomic Analysis). In addition, the Yale Center for Research Computing offers different tiers for storage and sharing data services in a secured manner (https://research.computing.yale.edu/services).

### Role of libraries

Although library-centric bioinformatics support programs cannot solve all of the challenges identified with personnel and training needs, they can be instrumental in helping ameliorate some of them. A few libraries are already supporting high-throughput data analysis by licensing user-friendly proprietary bioinformatics tools (Chattopadhyay et al., 2006; Li, Chen & Clintworth, 2013). An important point here is that, with most proprietary software, no programming skills are needed. In addition, companies provide technical support, which makes users feel safe (Smith, 2015). This approach is best for those with no command-line or scripting language skills for high-throughput data analysis. Regardless of whether a library can afford to license an actual product, however, librarians can invite and host vendors to demonstrate different commercial software products. These types of sessions can promote awareness of applicable tools and how to use them which researchers can choose to purchase if the library cannot.

Another strategy followed has been to partner with other centers and organizations on campus to provide training opportunities. Creating partnerships with other campus units and individuals (e.g., bioinformaticians, faculty) allows libraries to broaden and package data analysis services, including training opportunities. When training needs exceed library capacity, partnering with intramural and extramural units will be crucial in library support of health sciences bioinformatics research (Oliver, 2017).

As discussed above, there is a perceived need to learn R and scripting language. Many large academic institutions including Yale University have statistical consulting services, with staff who can provide coding classes in languages such as R, Unix, or Python. It is important, however, that these sessions are task-oriented and tailored to the specific needs of biomedical researchers who may not appreciate a basic class traditionally offered to wider campus constituents.

Since it may be difficult for bioinformaticians, who are in high demand to assist with primary research, to find time to provide formal training sessions for researchers, turning to peer-teachers, can be a positive and realistic alternative. Peer-teachers are individuals who are not official teachers in a particular field, but help those from a similar social group learn a particular skill set or specific concepts (Topping, 1996). Peer-teachers share similar experiences, challenges, and a set of knowledges as those they are educating, which allows them to effectively communicate the information they are teaching to students who are their peers. Therefore, they are better able to tailor the instruction material to the attendees’ current needs which can result in better learning outcomes (Yu et al., 2011).

Lastly, academic libraries can make it a strategic priority to build up their own bioinformatics programs. To do this successfully, library administration would need to devote more funding for commercial bioinformatics software to assist with routine analysis, and to hire librarians with backgrounds in bioinformatics who can identify and manage these resources and develop workshops and training sessions tailored to their researchers’ needs (Li, Chen & Clintworth, 2013). This investment, however, would give libraries a new relevancy to constituents who may not feel connected to the library as a service provider.

As this manuscript was being written, a newly created Yale Center for Biomedical Data Science was inaugurated with the goals of “build critical computational & informatics strength in a range of fields”, and “to connect experts and non-experts”. With these goals, it is easy to envisage that this center will help mitigate some of the challenges and needs found by this study.

## Limitations

One limitation of this study is its low response rate, which may introduce response bias. However, while 20% (176 out of 860) might seem low for survey research, previous web-based surveys of biomedical professionals reporting rates under 20% (and as low as 5%) are not uncommon.(Beck & Tieder, 2015; Golnik, Ireland & Borowsky, 2009; Rodriguez et al., 2006)

Another limitation is that because potential subjects were not offered any kind of incentive, it may be possible that those who agreed to participate did so out of interest in the subject matter, thus introducing the possibility of volunteer bias. Overall, we acknowledge that the present study may not represent the views of the entire biomedical community but it does highlight relevant topics and may provide initial insights into the needs and challenges of researchers dealing with high-throughput data analysis issues at Yale University and similar academic institutions.

## Conclusions

The growing amount of data generated by the application of high-throughput methodologies in biomedical research poses new challenges in terms of data analysis. The main challenges or barriers exposed by our study are:

• increased training needs for data analysis, including a perceived need to learn scripting language;
• perceived need of more personnel for data analysis;
• perceived inadequate communication between bioinformaticians and biomedical researchers.

In an academic environment, medical and/or science libraries may have a positive impact by establishing bioinformatics support programs that provide commercial bioinformatics tools for those with poor or no scripting language skills, and by organizing peer-teaching sessions to catalyze instruction on campus.

##  Supplemental Information

10.7717/peerj.5553/supp-1 Supplemental Information 1 Consolidated criteria for reporting qualitative research (COREQ) checklist

## Additional Information and Declarations

### Data Availability

The following information was supplied regarding data availability: Repository: Yale EliScholar Authors Interview transcripts https://works.bepress.com/rolando_garciamilian/31/. Survey data: Rolando Garcia-Milian. “Survey Data: Data Challenges of Biomedical Researchers in the Age of Omics” (2018) Available at: http://works.bepress.com/rolando_garciamilian/29/.
