# Supplementary material for: Data challenges of biomedical researchers in the age of omics
Source: PeerJ. 2018 Sep 11;6:e5553. doi: 10.7717/peerj.5553 (PMC6138043; doi:10.7717/peerj.5553)
Supplement: Supplemental Information 1 [file peerj-06-5553-s001.pdf]

Consolidated criteria for reporting qualitative studies (COREQ): 32-item checklist		
No	Item	Description
Domain 1: Research team and reflexivity		
Personal Characteristics		
1	Interviewer/facilitator	DH, RGM, FD
2	Credentials	DH MA, MLA/ RGM MLS, AHIP/ FD grad student
3	Occupation	Medical librarians
4	Gender	DH and FD female / RGM male
5	Experience and training	DH and RGM have previously conducted and published qualitative research. FD was trained by DH and RGM
Relationship with participants		
6	Relationship established	No
7	Participant knowledge of the interviewer	Interested in participating in the study
8	Interviewer characteristics	interest in the research topic
Domain 2: study design		
Theoretical framework		
9	Methodological orientation and Theory	Content analysis
Participant selection		
10	Sampling	Purposive
11	Method of approach	Email for contact - face to face for interview
12	Sample size	15
13	Non-participation	None
Setting		
14	Setting of data collection	workplace
15	Presence of non-participants	None
16	Description of sample	Representative of the main groups Faculty, Postocs, Grad Student
Data collection		
17	Interview guide	Questions based on questionnaire
18	Repeat interviews	None
19	Audio/visual recording	Audio-recording
20	Field notes	Transcription of the recordings
21	Duration	No more than 30 min each
22	Data saturation	Data saturation was discussed and avoided
23	Transcripts returned	Yes
Domain 3: analysis and findingsz		
Data analysis		
24	Number of data coders	DH / RGM/ FD
25	Description of the coding tree	Main themes
26	Derivation of themes	Themes derived from the data
27	Software	Nvivo 10
28	Participant checking	No
Reporting		
29	Quotations presented	Participants quotations presented to illustrate themes/findings. Each quotation identifies a representative of one of the main groups (e.g. faculty, postdoc)

30	Data and findings consistent	Data and findings were consistent
31	Clarity of major themes	Major themes are presented as headings
32	Clarity of minor themes	Only focused on major themes
